# Sudden Vision Loss in a Patient With Renal Cell Carcinoma: A Potential Indicator of Choroidal Metastasis

**DOI:** 10.7759/cureus.71138

**Published:** 2024-10-09

**Authors:** Nadja Kührer, Gertrud Haas, Hamed Wafa, Gerald Klinglmair, Renate Pichler

**Affiliations:** 1 Urology, Medical University of Innsbruck, Innsbruck, AUT; 2 Ophthalmology, Medical University of Innsbruck, Innsbruck, AUT

**Keywords:** case report, choroidal metastasis, metastatic, papillary renal cell cancer, rcc, targeted therapy

## Abstract

Choroidal metastasis of renal cell cancer (RCC) is an exceptionally rare clinical occurrence. In most cases, sudden vision loss is the first symptom. Here we present the case of a 52-year-old male with papillary RCC first diagnosed in 2018. Three years later, a bronchoscopy with endobronchial ultrasound (EBUS) identified suspicious infracarinal lymph nodes. A biopsy confirmed metastasis from RCC, leading to the initiation of systemic first-line therapy with cabozantinib monotherapy. The patient confirmed an excellent response with complete remission. In 2023, the patient reported for the first time a bilateral decrease in vision. Initial management with corrective lenses was unsuccessful. Further staging indicated metastases in the liver, spleen, and adrenal gland. Consecutively, the therapy was switched to lenvatinib and pembrolizumab. Two months later, an ophthalmologic examination due to persisting vision loss confirmed bilateral choroidal metastases. The systemic therapy was continued, and the patient's vision significantly improved. In 2024, the patient developed immune-associated pneumonitis, initially treated with prednisolone. The pulmonary situation became worse. A CT scan confirmed additional metastases in the lung with lymphangiosis carcinomatosis. Due to a poor performance status, no further systemic therapy has been initiated to date. In conclusion, this case highlights the rarity of choroidal metastases in RCC and the challenges of diagnosis. Ophthalmologic examination in RCC patients experiencing sudden vision loss is essential to detect these specific metastases as soon as possible. Comprehensive documentation and awareness of these rare metastatic manifestations are essential to improving diagnostic accuracy and patient outcomes.

## Introduction

Metastases from other primary tumors such as breast and lung cancer represent the most common form of malignant ocular lesions, with choroidal metastases being the most prevalent type due to the rich vascularization of the choroid [1]. Although metastases in well-vascularized areas of the eye are common, the eye remains a very rare localization of metastasis from renal cell carcinoma (RCC). To date, only 31 cases of choroidal metastasis of RCC are described in the literature [2]. Due to their rarity, these cases are still occasionally misdiagnosed. In most cases, sudden vision loss due to choroidal metastases was the first symptom and occurred before the primary diagnosis of RCC [3].

In this case report, we describe a patient diagnosed with papillary RCC who developed choroidal metastasis during the metastatic stage of the disease.

## Case presentation

We present the case of a 52-year-old male patient diagnosed in 2018 with a localized 19 mm left renal cell carcinoma (RCC) on computed tomography. Consecutively, the patient underwent a laparoscopic partial nephrectomy, confirming a papillary RCC grade I, stage pT1a with negative surgical margins (R0 resection). Follow-up examinations showed no evidence of recurrence. Three years postoperatively, bronchoscopy with endobronchial ultrasound (EBUS) revealed suspicious infracarinal lymph nodes. A biopsy with histological examination confirmed metastasis from RCC. The patient was classified as a favorable Memorial Sloan-Kettering Cancer Center (MSKCC) and International Metastatic RCC Database Consortium (IMDC) risk group. Consequently, the patient was started on systemic first-line (1L) therapy with cabozantinib 60 mg monotherapy in November 2021. Subsequent follow-up CT scans showed complete remission. In June 2022, the follow-up imaging revealed suspicious micronodular lesions in the left lung's lingula region. Close monitoring with CT every three months followed. The next imaging showed stable disease.

In May 2023, the patient first reported bilateral vision deterioration, initially managed by the general practitioner with corrective lenses but without therapeutic success. In July 2023, subsequent imaging revealed progressive disease with suspicious lesions in the adrenal gland, liver, and spleen. Pleural carcinomatosis is suspected. This prompted a switch to second-line therapy with lenvatinib 20 mg and pembrolizumab 400 mg. During the next follow-up visit, the patient reported a further decrease in vision. A cranial CT scan was performed, but it showed no evidence of intracranial masses, hemorrhage, or infarcts, and no imaging findings explained the patient’s vision loss. This raised suspicion of potential neurotoxic side effects from the systemic therapy. However, since the vision deterioration occurred before the last therapy change and did not improve an ophthalmologic examination was requested. The examination revealed bilateral choroidal metastases (L: n = 1; R: n = 4).

**Figure 1:**
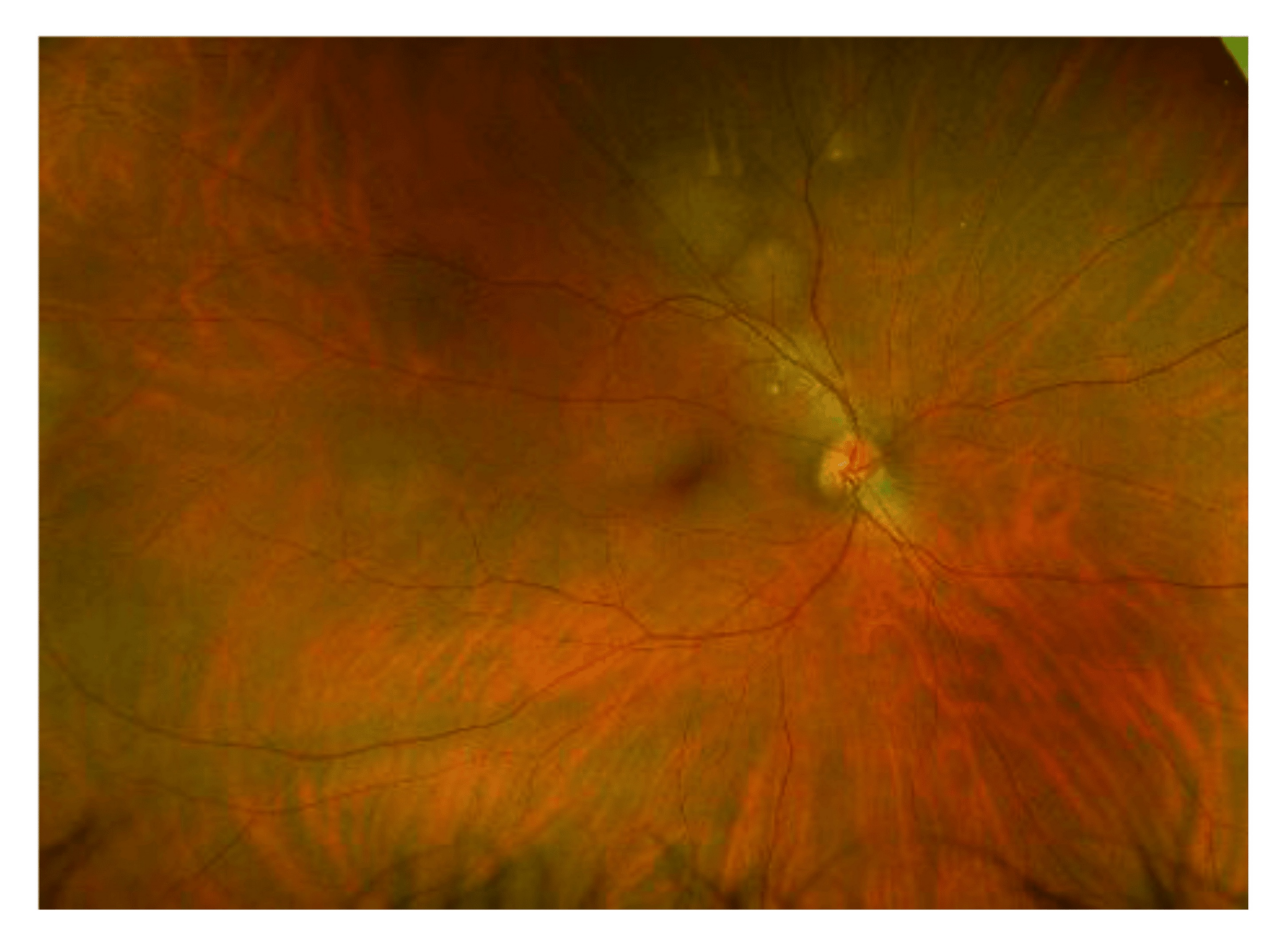
Right eye, multiple yellowish choroidal metastases

**Figure 2:**
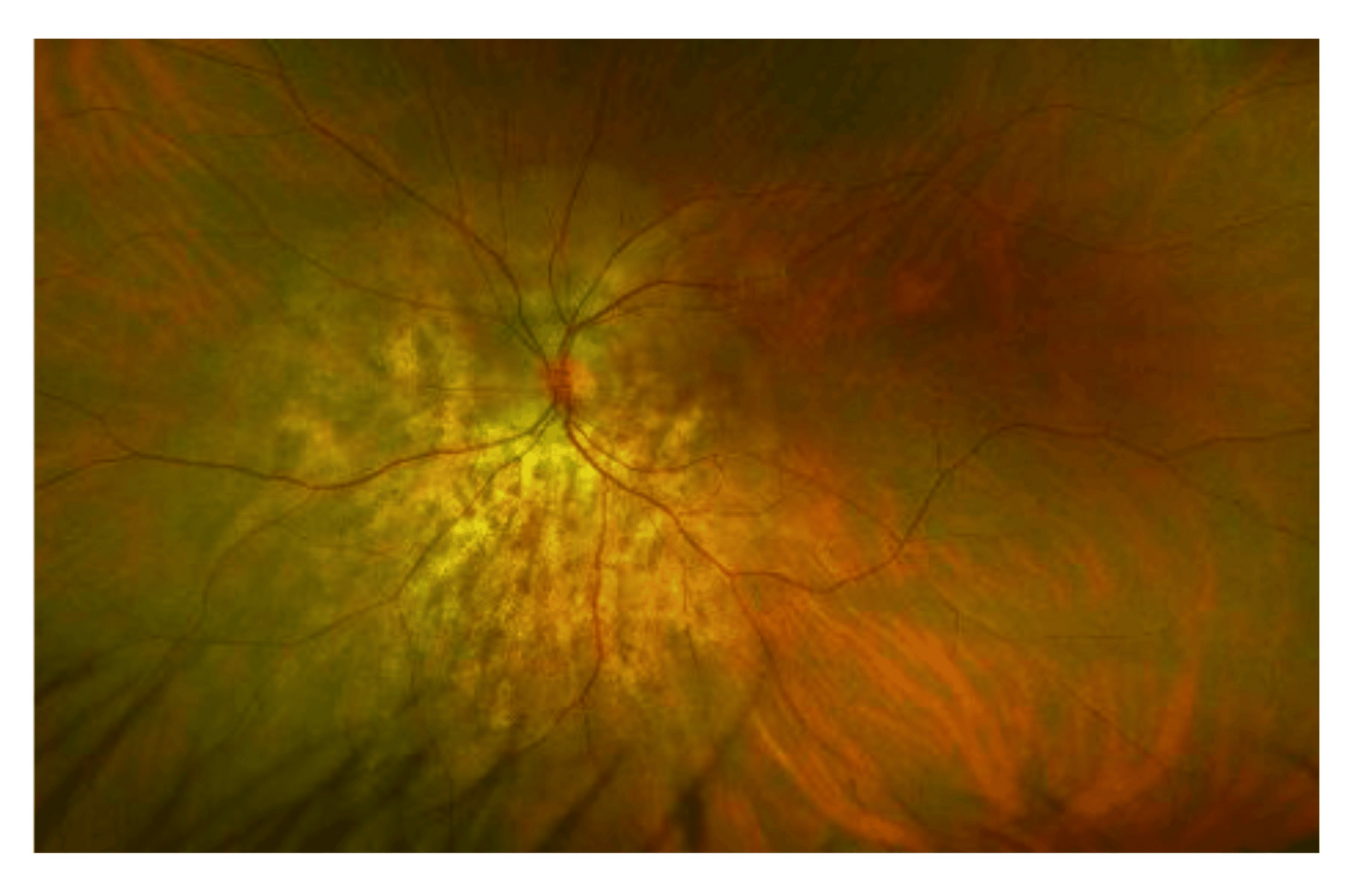
Left eye, large peripapillary choroidal metastasis

**Figure 3:**
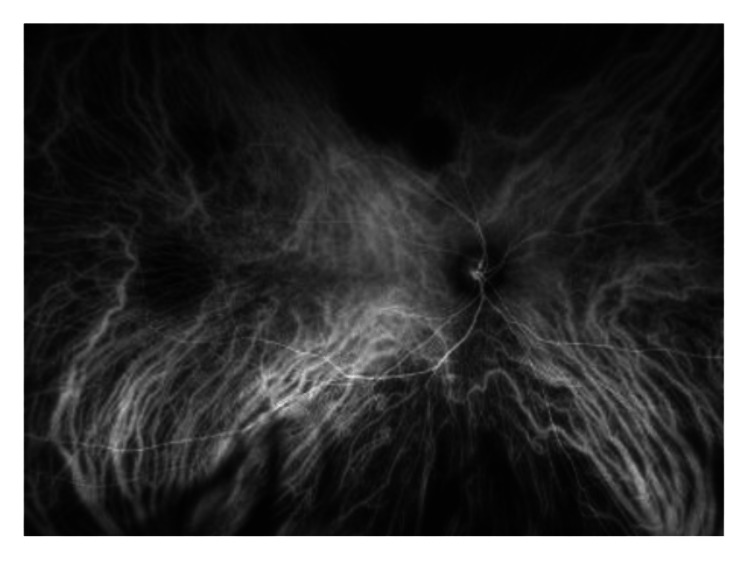
Indocyanine green angiography of the right eye with hypofluorescence

**Figure 4:**
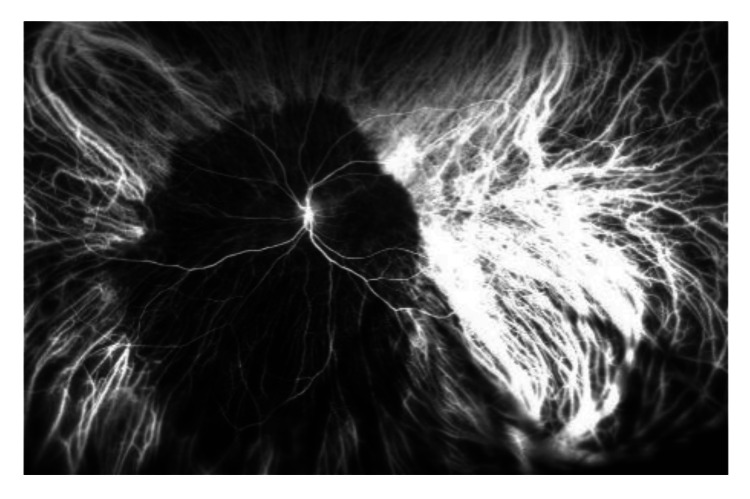
Indocyanine green angiography of the left eye with hypofluorescence

**Figure 5:**
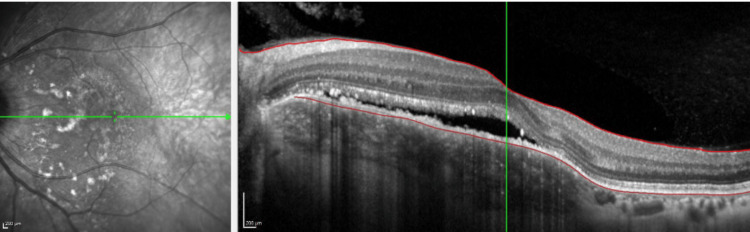
OCT of the left eye, choroidal prominence with subretinal fluid OCT: optical coherence tomography

There are multiple yellowish choroidal metastases in the right eye (Figure 1). On the left eye, there is another large peripapillary choroidal metastasis (Figure 2). In the early phase of indocyanine green angiographie, there is typical hypofluorescence (Figures 3, 4). In optical coherence tomography (OCT), there is a choroidal prominence with subretinal fluid, which is responsible for the blurred vision in the left eye (Figure 5).

**Figure 6:**
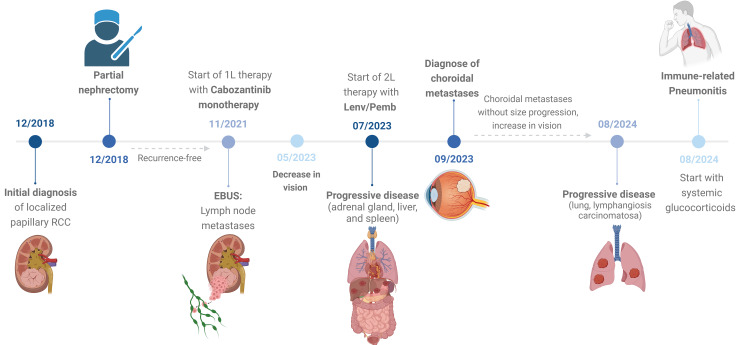
Timeline of patient history Figure created with Biorender.com RCC: renal cell cancer

The systemic therapy with lenvatinib 20 mg and pembrolizumab 400 mg was continued. The vision loss gradually stabilized after the therapy change. Subsequent ophthalmologic controls showed stable findings. In August 2024, imaging confirmed lung metastases with lymphangiosis carcinomatosa. At the same time, the patient developed immune-associated pneumonitis, which was treated with high doses of glucocorticoids (1 mg/kg of body weight). The patient's pulmonary condition continued to deteriorate. Due to the patient's poor performance status, no further systemic therapy is currently planned. An exact timeline of the patient's history is shown in Figure 6.

## Discussion

In this case report, we presented a patient with bilateral choroidal metastases of a papillary RCC, occurring four years after the initial diagnosis of the primary tumor. As reported in the literature, the reduction in visual acuity in our case was initially misdiagnosed and treated with corrective lenses [4-5]. Due to the rarity of choroidal metastases in RCC, the metastases were diagnosed later through an ophthalmologic examination. The continuous decrease in vision, despite corrective lenses, finally led to a referral to an ophthalmic center.

Only a few cases of choroidal metastases in RCC have been reported in the literature. The following table presents an overview of published cases of choroidal metastases from renal cell carcinoma (Table 1) [2,4-18].

**Table 1:** Overview of published data with choroidal metastases from renal cell cancer . Reference: [2,4-18] NOS: not otherwise specified; RCC: renal cell cancer

Author	Year	Sex	Age	Histological subtype of RCC	Time period between RCC and choroidal metastases	Latest therapy of (m) RCC	Therapy of choroid metastases
Emanuel and Guerra [6]	1974	M	50	NOS	NA	NA	NA
Stephens and Shields [7]	1979	M	NA	NOS	NA	NA	NA
Kindermann et al. [8]	1981	M	58	ccRCC	NA	NA	NA
Holbach et al. [9]	1990	M	75	ccRCC	9 years	NA	NA
Langmann and Müllner [10]	1994	M	56	NOS	6 months	Nephrectomy	Spontaneous regression
Haimovici et al. [11]	1997	M	54	NOS	Metastases before RCC diagnose	Nephrectomy	Proton beam irradiation
Haimovici et al. [11]	1997	M	62	NOS	Metastases before RCC diagnose	Nephrectomy	Radiation therapy
Haimovici et al. [11]	1997	M	48	NOS	9 years	Systemic chemotherapy, NOS	Systemic chemotherapy, NOS
Haimovici et al. [11]	1997	F	66	ccRCC	6 years	Nephrectomy	Proton beam irradiation
Haimovici et al. [11]	1997	M	77	NOS	7 years	Nephrectomy	Enucleation of the eye
Shields et al. [12]	1997	8 patients, mean 65 y		NOS	NA	NA	NA
Srinivasan and Gray [13]	2003	38	M	ccRCC	2 months	Radiation therapy	Radiation therapy
Hammad et al. [14]	2003	48	F	NOS	Metastases before RCC diagnose	Nephrectomy	Spontaneous regression
Bellerive et al. [4]	2016	73	M	ccRCC	25 years	Nephrectomy, Sunitinib (first-line)	Enucleation of the eye
Essadi et al. [15]	2017	62	M	ccRCC	2.5 years	Everolimus (second-line)	Everolimus (second-line)
Ayres et al. [16]	2017	81	M	ccRCC	9 years	Brachytherapy	NA
Komanski et al. [5]	2017	73	M	ccRCC	4 years	Nephrectomy	Enucleation of the eye (second-line)
Chao et al. [17]	2021	50	M	ccRCC	1 year	NA	Targeted therapy, NOS (first-line)
Xu et al. [18]	2021	45	F	NOS	1 year	Nephrectomy	None
Hiruta et al. [2]	2024	60	M	pRCC, type 2	7 years	Pembrolizumab/Axitinib (first-line)	Radiation therapy
Hiruta et al. [2]	2024	76	M	pRCC, type 1	NA	Axitinib (second-line)	Axitinib (second-line)

As shown in Table 1, most choroidal metastases arise from clear cell RCC [2,4-18]. This might be explained by the fact that clear cell RCC is the most common histological subtype of RCC in 80% [19].

Historically, treatment options for choroidal metastases were limited and primarily included local interventions such as conventional radiotherapy, proton radiation, or enucleation of the affected eye, particularly in cases published before 1997 [6-11]. However, recent advancements in systemic therapy have been groundbreaking with various combinations in the first-line mRCC therapy consisting of nivolumab/ipilimumab [20], pembrolizumab/axitinib [21], nivolumab/cabozantinib [22], and pembrolizumab/lenvatinib [23].

In our case, the initial treatment with cabozatinib monotherapy in 2021 was later switched to the pembrolizumab/lenvatinib combination in July 2023 due to disease progression. This combination was associated with notable success in managing the choroidal metastases, resulting in improved visual acuity and stabilization of the ocular lesions, with no new metastases detected. Thus, choroidal metastases appear to respond not only to local therapy but also to modern systemic therapy. Literature indicates a wide variability in the time from primary tumor diagnosis to the development of choroidal metastases, ranging from two months to 25 years [4,13]. In our case, choroidal metastasis occurred four years after the primary diagnosis of RCC. The significant advancements of highly efficient new systemic therapies, such as vascular endothelial growth factor receptor (VEGFR)-targeted therapy and immune checkpoint inhibitors, have significantly improved survival rates, underscoring the importance of monitoring for rare metastatic sites like the choroid [24].

## Conclusions

Choroidal metastases from RCC are exceptionally rare, which often leads to misdiagnosis. Due to the infrequent occurrence of such metastases, awareness among clinicians is crucial. Comprehensive documentation of these cases can significantly contribute to recognizing this rare metastatic localization, highlighting the clinical importance of an ophthalmologic examination in patients who experience a sudden decrease in vision. A yearly eye examination as a screening should be considered. This may help to reduce cases of misdiagnosis and avoid unnecessary treatment delays, ultimately improving patient outcomes.
